# Renal Function Can Improve at Any Stage of Chronic Kidney Disease

**DOI:** 10.1371/journal.pone.0081835

**Published:** 2013-12-13

**Authors:** Lise Weis, Marie Metzger, Jean-Philippe Haymann, Eric Thervet, Martin Flamant, François Vrtovsnik, Cédric Gauci, Pascal Houillier, Marc Froissart, Emmanuel Letavernier, Bénédicte Stengel, Jean-Jacques Boffa, on behalf of the NephroTest Study Group

**Affiliations:** 1 Department of Nephrology, AP-HP, Hôpital Tenon, Paris, France; 2 Research Centre in Epidemiology and Population Health, Inserm Unit 1018, CESP, Villejuif, France; 3 UMRS 1018, Univ Paris-Sud, Villejuif, France; 4 Department of Physiology, AP-HP, Hôpital Tenon, Paris, France; 5 INSERM UNIT 702, Paris, France; 6 UMR S 702, Univ Pierre et Marie Curie-Paris 6, Paris, France; 7 Department of Nephrology, AP-HP, Hôpital Européen Georges Pompidou, Paris, France; 8 UMR S 775, Univ Paris Descartes, Paris, France; 9 Department of Physiology, AP-HP, Hôpital Bichat, Paris, France; 10 Department of Nephrology, AP-HP, Hôpital Bichat, Paris, France; 11 Department Physiology, AP-HP, Hôpital Européen Georges Pompidou, Paris, France; Mario Negri Institute for Pharmacological Research and Azienda Ospedaliera Ospedali Riuniti di Bergamo, Italy

## Abstract

**Introduction:**

Even though renal function decline is considered relentless in chronic kidney disease (CKD), improvement has been shown in patients with hypertensive nephropathy. Whether this can occur in any type of nephropathy and at any stage is unknown as are the features of patients who improve.

**Methods:**

We identified 406 patients in the NephroTest cohort with glomerular filtration rates (mGFR) measured by ^51^Cr-EDTA clearance at least 3 times during at least 2 years of follow-up. Individual examination of mGFR trajectories by 4 independent nephrologists classified patients as improvers, defined as those showing a sustained mGFR increase, or nonimprovers. Twelve patients with erratic trajectories were excluded. Baseline data were compared between improvers and nonimprovers, as was the number of recommended therapeutic targets achieved over time (specifically, for systolic and diastolic blood pressure, proteinuria, and use of renin angiotensin system blockers).

**Results:**

Measured GFR improved over time in 62 patients (15.3%). Their median mGFR slope was +1.88[IQR 1.38, 3.55] ml/min/year; it was −2.23[−3.9, −0.91] for the 332 nonimprovers. Improvers had various nephropathies, but not diabetic glomerulopathy or polycystic kidney disease. They did not differ from nonimprovers for age, sex, cardiovascular history, or CKD stage, but their urinary albumin excretion rate was lower. Improvers achieved significantly more recommended therapeutic targets (2.74±0.87) than nonimprovers (2.44±0.80, p<0.01). They also had fewer CKD-related metabolic complications and a lower prevalence of 25OH-vitamin-D deficiency.

**Conclusion:**

GFR improvement is possible in CKD patients at any CKD stage through stage 4–5. It is noteworthy that this GFR improvement is associated with a decrease in the number of metabolic complications over time.

## Introduction

Chronic kidney disease (CKD), which is associated with an increased risk of morbidity and mortality, is becoming increasingly common. Once CKD has developed, the glomerular filtration rate (GFR) declines at a rate that is considered inexorable albeit highly variable. Furthermore, patients whose GFR declines rapidly are at a particularly high risk of adverse cardiovascular disease events [1]. Longitudinal studies of CKD patients have identified both modifiable and non-modifiable risk factors of CKD progression, including age, sex, race, hypertension, urinary albumin excretion (UAE), diabetes, low HDL-cholesterol, and the underlying cause of nephropathy [2]–[5]. Interventional studies have emphasized the beneficial effects of angiotensin 2 blockers (ACE inhibitors and angiotensin receptor blockers) in reducing systemic and glomerular pressure and urinary albumin excretion and demonstrated their ability to delay end stage renal disease (ESRD) [6]–[8]. The US National Kidney Foundation has developed guidelines to promote early detection of CKD and thus prevent its progression and complications [9]. Nonetheless, renal function still declines in most CKD patients [10]–[12]. Studies are investigating modulation of new pathophysiological pathways to delay or even reverse CKD progression [13]–[17].

Although most CKD patients progressively lose renal function, a few studies have reported a significant proportion of patients with stable or improved renal function over time. Investigators for the REIN Follow-Up study showed that prolonged ACE inhibition can induce remission even in patients with severe disease [18]. The MDRD study found that although GFR declined over time in 85% of patients, it increased in 15% [3]. Using the data from the African-American Study of Kidney Disease and Hypertension (AASK) trial, Hu *et al* reported improvement in kidney function among a subset of patients with hypertensive CKD [19]. As in HIV infection, there are lessons to be learned from long-term resilience to disease progression in CKD [20]. Whether this can occur in any type of nephropathy and at any stage is unknown. Moreover, the patient features associated with sustained GFR improvement are not yet well documented.

Our aim in this study was to identify patients in the NephroTest cohort whose repeated GFR measurements over time showed an improvement in renal function over time and to describe their characteristics. This ongoing prospective cohort includes patients with various nephropathies and all stages of CKD except dialysis. We compared this subgroup with the rest of the cohort to identify factors associated with better prognosis.

## Methods

### Cohort Patients

**Figure 1:**
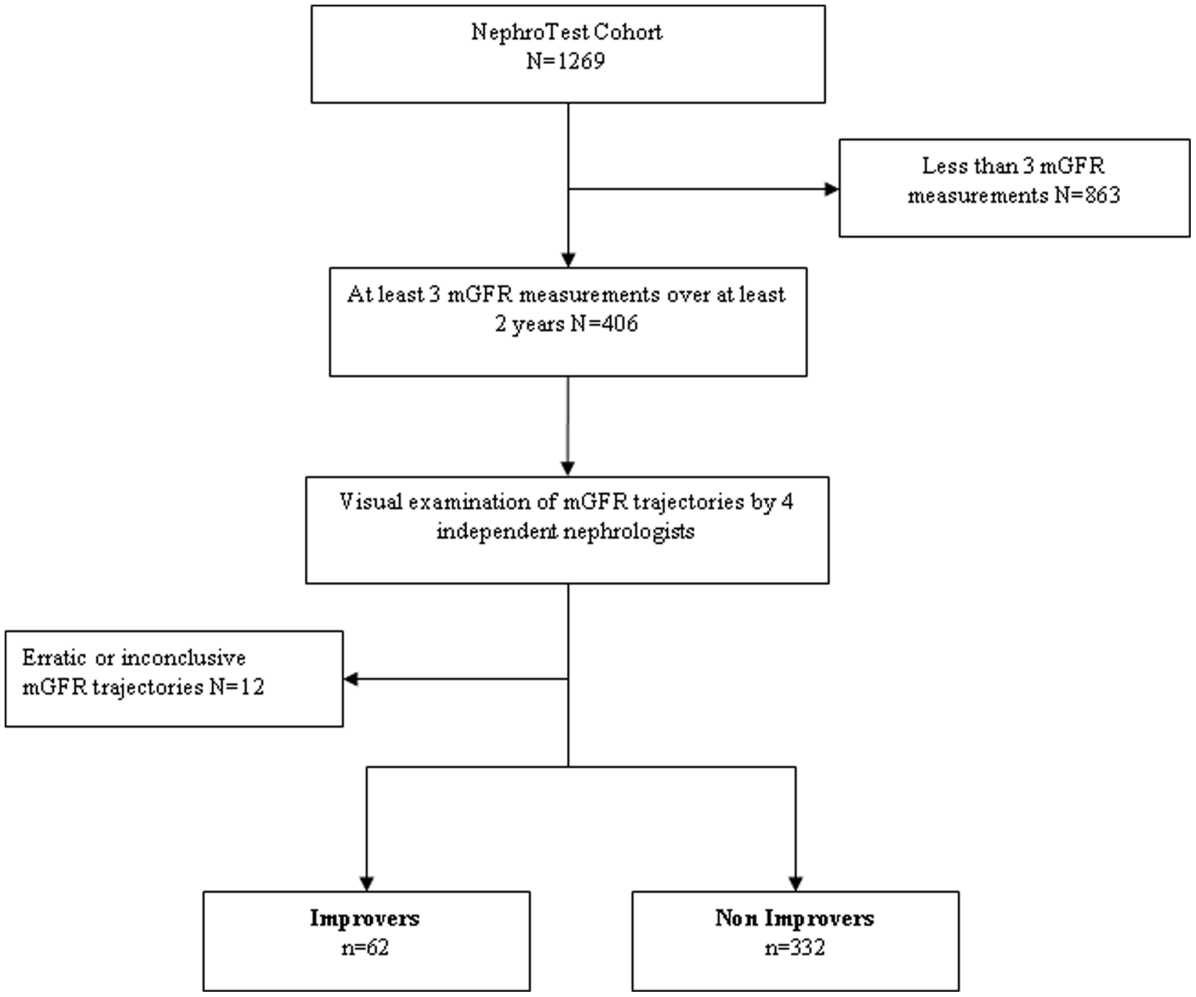
Study flow chart.

The NephroTest study is an ongoing prospective hospital-based cohort, enrolling patients with any diagnosis of CKD stages 1 through 5. Two nephrology departments recruited these patients, who undergo extensive annual work-ups, including measured GFR (mGFR), in the hospitals’ physiology departments [21]. Patients younger than 18 years, those on dialysis or with a kidney transplant, and pregnant women are excluded. Between January 2000 and January 2008, 1269 patients were included after providing informed consent. We excluded 621 patients with only one mGFR and 242 with two and studied 406 patients with at least three mGFR measurements during at least two years of follow- (Figure 1).

### Classification of Patients According to Trend Over Time of Renal Function

As reported elsewhere, all patients had GFR measured by ^51^Cr-EDTA renal clearance [21]. Improvers were not defined solely by a positive GFR slope. Indeed, because slopes estimated by linear regression tend to be more variable than the true slopes, such a method might well have overestimated the proportion of improvers. Improvers were thus defined qualitatively, based on individual examination of all mGFR trajectories by four independent nephrologists. After excluding 12 patients with erratic or inconclusive curves, defined as patients alternating increased and decreased mGFR without a clear trend, they classified those whose mGFR increased sustainably over time as improvers, and the others as nonimprovers (Figure S1 in File S1, supplemental data). When two or more nephrologists disagreed about a patient’s status, consensus was reached before analysis. The Kappa statistic showed excellent agreement among the four nephrologists for all patients (kappa = 0.76) and for the subgroup of improvers (kappa = 0.80).

### Data and Measurements

Data recorded during annual 5-h visits to one of the two hospital physiology departments included demographics, primary renal diagnosis, medical history, height and weight, resting blood pressure (BP), and medications. Diabetes was either self-reported or defined as fasting glycemia>7 mmol/L or antidiabetic drug treatment. Smoking was self-reported. Cardiovascular disease history was defined as a history of stroke, ischemic heart disease (angioplasty, surgical coronary bypass, or myocardial infarction), or heart failure. The causes and courses of nephropathies were verified from each patient’s original medical file.

The mGFR was assessed by ^51^Cr-EDTA renal clearance as previously described [22]. Briefly, 1.8 to 3.5 MBq of ^51^Cr-EDTA (GE Healthcare, Velizy, France) was injected intravenously as a single bolus. Average renal ^51^Cr-EDTA clearance was then determined over 5 to 6 consecutive 30-min clearance periods. The coefficient of variation was 8.4% ±5.0 (n = 22). GFR was also estimated by the MDRD and CKD-EPI equations, with IDMS-traceable plasma creatinine values. We measured parathyroid hormone (PTH) concentration by a second-generation two-site immunoradiometric assay (ELSA-PTH kit from Cisbio Bioassays), plasma phosphate concentration by colorimetry (phosphomolybdate assay), calcium serum levels by flame photometry (Perkin Elmer 3300 atomic absorption spectrometer), potassium serum levels and venous total CO_2_ (tCO_2_) with a specific electrode (ABL 815 from Radiometer and Beckman SX9), and albuminemia by immunonephelometry. We measured urinary protein by colorimetry (pyrogallol red with molybdate) and expressed the results as the urinary protein-creatinine ratio (UPCR) mg/mmol creatinine. Urinary albumin was measured with solid-phase fluorescent immunoassays and expressed as the urinary albumin-creatinine ratio (UACR). Plasma 25(OH)D concentration was measured by a radioimmunological method that recognized both 25OHvitamin D_2_ and D_3_. Normal values range from 10 to 40 pg/ml.

Thresholds used to define metabolic complications were based on current international guidelines whenever available. We used K/DOQI criteria to define anemia, that is, Hb concentration <110 g/l. We also calculated anemia prevalence estimates based on WHO gender-specific thresholds: Hb concentration <130 g/l for men and <120 g/l for women. Hyperparathyroidism was defined as an intact PTH concentration >60 pg/ml (laboratory reference values 10 to 60 pg/ml), hyperphosphatemia as a plasma phosphate value >4.3 mg/dl (or 1.38 mmol/L), metabolic acidosis as a tCO_2_ value <22 mmol/l, hyperkalemia as serum potassium >5 mmol/l, and 25(OH)D deficiency as <15 ng/ml.

### Number of Achieved Therapeutic Targets

We studied four therapeutic targets: systolic and diastolic blood pressure: <130 mm Hg and <80 mm Hg respectively, albuminuria <3 mg/mmol of creatinine or proteinuria <20 mg/mmol, and use of renin angiotensin system blocker. We assigned 1 point if the therapeutic target was achieved, 0 if it was not achieved.

### Statistical Analysis

The Kappa statistic estimating agreement among the four nephrologists was computed withthe SAS macro %MAGREE ([23]. We then used linear regression to estimate individual mGFR and eGFR slopes. The difference from zero of median mGFR slopes was tested with theWilcoxon signed rank test. To assess the phenomenon of regression to the mean, we performed two sensitivity analyses: mGFR slopes were estimated (i) using the mean of the first and second mGFR as the baseline mGFR, (ii) without considering baseline mGFR values. Differences between mGFR slopes estimated using all mGFR measurements and either of these two methods were tested with the Wilcoxon signed rank test.

Clinical and laboratory data are expressed as percentages, with means (± sd) or medians (interquartile range, IQR) reported, as appropriate. We compared baseline clinical and laboratory data and cardiovascular and CKD risk factors between improvers and nonimprovers. The sample size for each variable is specified in the tables. Continuous variables were compared with the Wilcoxon test and categorical variables with the chi2 or Fisher’s exact test. Linear or logistic regression allowed us to test whether adjustment for mGFR affected any significant crude differences found between the groups.

We compared improvers and nonimprovers for the number of therapeutic targets achieved at baseline and at study end and for the mean over time. We then performed a logistic regression analysis to estimate of kidney function improvement according to the number of therapeutic targets achieved at baseline or during follow up, adjusted for baseline or mean mGFR, center, age, gender, BMI, African origin, and diabetes. We studied mGFR improvement according to proteinuria and BP considered continuously (at baseline or using mean values over time) or as categorical variables (at baseline), adjusting for the same covariates and use of renin angiotensin system blocker.

Finally, we compared the type and number of antihypertensive medications, as well as the prevalence andnumber of CKD metabolic complications including hyperparathyroidism (PTH >60 ng/mL), anemia (Hb <110 g/L), hyperphosphatemia (phosphate >1.38 mmol/L), acidosis (venous tCO_2_ <22 mmol/L), hyperkalemia (potassium >5 mmol/L) at baseline and during follow-up between the two groups. Statistical analyses were performed with SAS 9.2 (SAS Institute, Cary, NC) and R (R Development Core Team, 2009).

## Results

### Group Characteristics

Sixty-two (15.3%) patients were classified as improvers, and 332 as nonimprovers. The median(IQR) mGFR slope for the improvers was 1.74[1.25,3.21] ml/min/year and for the nonimprovers, −2.31[−4.18, −1.02], both significantly different from zero (Table 1). It is worth noting that 36 (8.9%) patients with positive slopes were classified as nonimprovers by the four nephrologists (Figure S2 in File S1). For both improvers and nonimprovers, the measured GFR slopes calculated with all GFR measurements did not differ significantly from those estimated (i) by using the mean of the first and second mGFR as the baseline mGFR or (ii) without considering baseline mGFR values (Table 1). Improvers and nonimprovers had a median of 4 [min 3; max 8] mGFR measurements. Time between two consecutive mGFR measurements did not differ between improvers and nonimprovers, with a median time (IQR) of 12.6 (11.9–16.3) and 12.5 (11.8–14.5) months, respectively.

**Table 1:** Median slopes of measured and estimated GFR for improvers and non-improvers.

	N		median (IQR)
**Improvers**	62	mGFR slope (ml/min/yr)	1.74[1.25,3.21]
		without baseline mGFR values	1.96[1.10,3.51]
		with baseline mGFR values as the mean of 1^st^ and 2^nd^ mGFR	2.06[1.16,3.25]
		mGFR slope (ml/min/1.73 m^2^/yr)	1.88[1.38,3.55]
		eGFR CKD-EPI slope (ml/min/1.73 m^2^/yr)	2.04[0.81,4.26]
		eGFR MDRD slope (ml/min/1.73 m^2^/yr)	2.11[0.87,4.27]
		Creatinine clearance slope (ml/min/yr)	2.61[−0.73,6.08]
**Non Improvers**	332	mGFR slope (ml/min/yr)	−2.31[−4.18, −1.02]
		without baseline mGFR values	−2.34[−5.02, −0.56]
		with baseline mGFR values as the mean of 1^st^ and 2^nd^ mGFR	−2.36[−4.46, −0.99]
		mGFR slope (ml/min/1.73 m^2^/yr)	−2.23[−3.9, −0.91]
		eGFR CKD-EPI slope (ml/min/1.73 m^2^/yr)	−1.25[−2.87,0.08]
		eGFR MDRD slope (ml/min/1.73 m^2^/yr)	−1.10[−2.63,0.16]
		Creatinine clearance slope (ml/min/yr)	−2.33[−5.05,0.09]

The initial nephropathy among the 62 improvers was glomerular disease for 19, tubulo-interstitial nephropathy for 16, vascular nephropathy for 17 (15 nephroangiosclerosis and 2 thomboticmicroangiopathy) including diabetes for 9, nephron reduction for 6 (3 with nephrectomies for kidney neoplasms, 1 with congenital renal hypoplasia, and 2 with a single functional kidney), and undetermined for 4. Most patients with glomerular disease had had a renal biopsy (16/19). Interestingly, no improver had either diabetic glomerulopathy or polycystic kidney disease, diseases that affected 12.7% and 4.5% of the nonimprovers.

At baseline, the two groups did not differ significantly for age, sex ratio, ethnicity or BMI. Around 10% the patients had African ancestry. Both MDRD and CKD-EPI eGFR were significantly higher in improvers than nonimprovers, but the difference in median mGFR value was on the borderline of significance (Table 2). Of note, the analysis of the distribution of patients according to the K-DOQI/K-DIGO CKD staging showed that up to 24.2% of improvers had stage 4 or 5 CKD (Table 2).

**Table 2:** Baseline patient characteristics and kidney function according to CKD progression status.

Baseline characteristics	Improvers(n = 62)	Non improvers(n = 332)	p-value
Men	71.0 (44/62)	72.0 (239/332)	0.9
Age	58.4±14.5	58.5±14.6	0.9
African origin	12.1 (7/58)	8.5(27/316)	0.4
Body mass index	25.8±4.1	26.4±4.8	0.4
**Kidney function**			
mGFR, ml/min/1.73 m^2^	38.3[31.7–48.0]	34.6[25.9–45.7]	0.06
<15	1.6 (1/62)	3.3 (11/332)	
15–30	22.6 (14/62)	33.4 (111/332)	
30–45	45.2 (28/62)	37.4 (124/332)	
45–60	22.6 (14/62)	16.0 (53/332)	
>60	8.1 (5/62)	9.9 (33/332)	
eGFR MDRD, ml/min/1.73 m^2^	39.0[29.5–50.7]	31.8[24.6–42.7]	0.002
eGFR CKD-EPI, ml/min/1.73 m^2^	42.0[30.0–52.8]	32.5[24.9–45.3]	0.002

Abbreviations: mGFR, measured glomerular filtration rate; eGFR MDRD, estimated GFR using Modification of the Diet in Renal Disease equation; eGFR CKD-EPI, estimated GFR using Chronic Kidney Disease Epidemiology Collaboration equation.

### Cardiovascular and CKD Progression Risk Factors

Improvers had diabetes mellitus less often than nonimprovers (Table 3). The proportion of insulin-requiring patients and of glycated hemoglobin (HbA1C) values did not differ between the two groups. Smoking, history of cardiovascular disease, and lipids did not differ significantly across groups, but a smaller proportion of improvers than of nonimprovers received statin treatment.

**Table 3:** Baseline cardiovascular and renal risk factors according to CKD progression status.

Baseline characteristics	Improvers(n = 62)	Non improvers(n = 332)	p-value
Diabetes mellitus	14.5 (9/62)	26.2 (87/332)	0.05
Blood pressure (N = 61/322)			
Systolic	128 [117–138]	135 [122–149]	0.01
Diastolic	71 [67–79]	75 [68–84]	0.04
Mean	92 [84–98]	95 [87–105]	0.01
Pulse pressure	54 [46–65]	58 [51–70]	0.04
Antihypertensive treatments	98.2 (55/56)	95.3 (307/322)	0.5
ACEi or ARB	73.2 (41/56)	82.6 (266/322)	0.1
Loop diuretics	35.7(20/56)	40.4 (130/322)	0.5
Number of different medications	2.3±1.2	2.6±1.3	0.2
**Cardiovascular risk factors**			
BP >140/90	24.6 (15/61)	41.9 (135/322)	0.01
Smoker	9.7 (6/62)	14.2 (47/332)	0.3
History of cardiovascular disease	14.5 (9/62)	15.4 (51/332)	0.9
Antiplatelet agents	26.8 (15/56)	31.4 (101/322)	0.5
Total cholesterol (mmol/L)	5.3±1.2	5.0±1.1	0.1
HDL (mmol/L)	1.3±0.5	1.3±0.4	0.8
LDL (mmol/L)	3.3±1.0	3.0±1.0	0.09
Hypercholesterolemia (LDL >2.6 mmol/L)	25.8 (16/62)	33.2 (106/319)	0.3
Hypolipidemic agents	42.9 (24/56)	52.2 (168/322)	0.2
* Statins*	28.6 (16/56)	45.7 (147/322)	0.02
**Risk factors for CKD progression**			
BP >130/80	42.6 (26/61)	59.6 (192/322)	0.01
UPCR mg/mmol (n = 49–303)	21.3 [12.1–33.4]	44.0 [17.1–114.0]	0.0002
UPCR >30 mg/mmol	30.6 (15/49)	57.1(173/303)	0.0006
UACR mg/mmol	4.4 [1.8–8.7]	12.1 [3.4–46.6]	<0.0001
Urea excretion/weight, mmol/l/kg	0.99±0.28	1.05±0.33	0.4
In patients with diabetes			
Insulin	33.3 (3/9)	56.3 (49/87)	0.3
HbA1c (%)	7.1±1.6	7.1±1.3	0.9

BP: blood pressure, UPCR: urinary protein-creatinine ratio, UACR: urinary albumin-creatinine ratio.

Improvers had lower systolic, diastolic, mean, and pulsed arterial BP than nonimprovers. Moreover, improvers had high BP, whether defined as >140/90 mmHg or 130/80 mmHg, less often than nonimprovers. At baseline, the proportion of patients receiving antihypertensive therapy, higher than 90%, did not differ between the two groups, nor did the mean number or type of medications. However, at the last visit, improvers used ACEi or ARBs and diuretic loops significantly less often, as well as significantly fewer types of different antihypertensive medications than nonimprovers (Table S1 in File S1).

Improvers had less urinary protein excretion than nonimprovers (Table 3) according to either UPCR or UACR. These differences persisted after adjustment for mGFR (UPCR p_adj_ = 0.0002, UACR p_adj_<0.0001). The daily protein intake, estimated from urinary excretion of urea nitrogen per kilogram of body weight, was similar between groups, as was the proportion treated with anti-platelet agents (aspirin or clopidogrel).

### Achieved Therapeutic Targets

Compared with nonimprovers, improvers achieved more therapeutic targets at both first and last visits (Table 4) and had a higher mean number of targets achieved across all visits. Crude odds ratio (CI 95%) of mGFR improvement according to each increase of one achieved therapeutic target was 1.34(1.04, 1.73) and 1.61(1.12, 2.30) for baseline and mean values, respectively. After adjusting for either baseline or mean mGFR, and for other covariates, the odds ratio of mGFR improvement remained significantly associated with the number of achieved therapeutic target for baseline values, 1.37(1.05, 1.79), and was slightly attenuated for mean values, 1.47(0.99, 2.18). Moreover, when including each therapeutic target into the logistic model with other covariates, patients without proteinuria (ACR<3 mg/mmol or PCR<20 mg/mmol) or with controlled blood pressure at baseline (systolic BP<130 mm Hg and diastolic BP<80 mm Hg) had significantly higher adjusted ORs of mGFR improvement : 2.41 (1.27, 4.57) and 1.93 (1.03, 3.60), respectively. Finally, when analyzing proteinuria and BP as continuous variables, mGFR improvement significantly increased with decreasing log proteinuria using either baseline or mean values, 0.54 (0.39, 0.75) and 0.61 (0.43, 0.86), respectively, but blood pressure (either mean or systolic or dialostic BP) was not, whether at baseline or for mean values.

**Table 4:** Mean (±sd) number of achieved thepareutic targets at first and last visit, and mean across visits, according to CKD progression status.

	Improvers(n = 62)	Non improvers(n = 332)	p-value
First visit	2.69±1.18	2.33±1.13	0.02
Last visit	2.69±0.97	2.41±1.00	0.03
Mean across visit	2.74±0.87	2.44±0.80	0.009

### CKD Complications

The proportion of patients with anemia, hyperkalemia, acidosis, or hypocalcemia did not differ between groups (Table 5). Hyperphosphatemia was uncommon, and its prevalence did not differ between the groups after adjustment for baseline mGFR. After adjustment for baseline mGFR, vitamin D deficiency was less common in improvers than in nonimprovers (p<0.001), and importantly this association persisted after additional adjustment for uACR (p = 0.02). PTH levels were significantly lower in improvers than in nonimprovers, but this association was no longer significant after adjustment for mGFR (p = 0.08).

**Table 5:** Biomarkers of metabolism disorders and treatment use according to CKD progression status.

Baseline characteristics	Improvers(n = 62)	Non improvers(n = 332)	p-value
Serum albumin, g/l	40.8±4.3	40.2±4.6	0.6
Serum albumin <38 g/l	20.0 (12/60)	28.2(91/323)	0.2
Hb (g/dL)	12.8±1.6	12.5±1.5	0.06
Anemia WHO definition‡	44.3 (27/61)	57.2(187/327)	0.06
Anemia KDOQI definition‡	11.5 (7/61)	15.6(51/327)	0.4
EPO use	5.4 (3/56)	3.4(11/322)	0.4
Plasma potassium (mmol/L)	4.19±0.48	4.34±0.50	0.07
[K+] >5 mmol/L	6.5 (4/62)	8.4(28/332)	0.6
**Mineral and Bone Metabolism**			
25(OH)D (ng/mL)	26.0 [15.7–32.9]	18.5 [11.0–28.4]	0.004
Deficiency (<15 ng/mL)	20.0 (12/60)	39.2(127/324)	0.005
1.25(OH)_2_D (pg/mL)	24.5 [18.5–32.0]	22.1 [15.5–30.0]	0.1
Ionised calcium (mmol/L)	1.22±0.07	1.21±0.07	0.2
Ca ion<1.10 mmol/L	3.2 (2/62)	2.7(9/331)	0.7
PTH (ng/mL)	49 [37–85]	70 [38–105]	0.03
PTH >60 ng/ml	40.7 (24/59)	56.9(185/325)	0.02
Serum phosphate (mmol/L)	1.04±0.19	1.12±0.22	0.02
Serum phosphate >1.38 mmol/L	3.2 (2/62)	11.1(37/332)	0.06
Phosphocalcic regulator treatment	26.8 (15/56)	23.6(76/322)	0.6
25(OH)D or analogues	7.1 (4/56)	11.2(36/322)	0.4
1,25(OH)D_3_ or analogues	8.9 (5/56)	7.1(23/322)	0.6
Calcium	21.4 (12/56)	15.5(50/322)	0.3
Phosphate chelators	3.6 (2/56)	0.9(3/322)	0.2
**Acid-base metabolism**			
Venous tCO_2_ (mmol/L)	26.27±2.99	25.57±3.00	0.04
venous tCO_2_<22 mmol/L	8.2 (5/61)	11.9(39/328)	0.4
sodium bicarbonate	0.0 (0/56)	4.3(14/322)	0.1
venous tCO_2_<22 mmol/L or sodium bicarbonate use	9.1 (5/55)	15.4(49/318)	0.2

Mean ± SD, median ± IQR, % (n).

Anemia according to WHO definition Hb <13 g/dL in men and <12 g/dL in women, KDOQI definition Hb <11 g/dL. PTH: parathyroid hormone, 25(OH)D and 1,25(OH)_2_D 1,25∶25-hydroxy- and 1,25-dihydroxy-vitamin D, Hb: hemoglobin, EPO: Erythropoietin.

**Figure 2:**
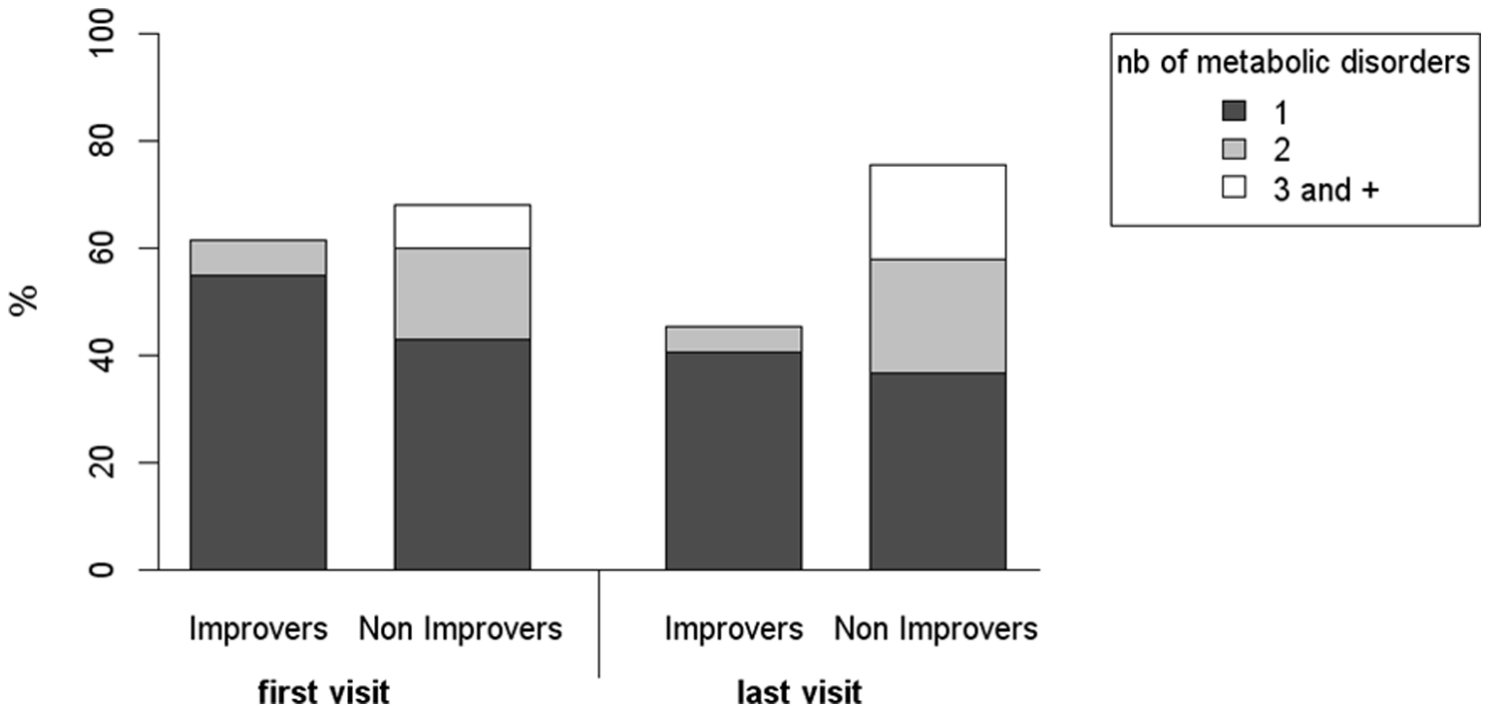
Percentage of patients with one, two, or three or more metabolic complications at first and last visit according to CKD progression status. Metabolic complications include hyperparathyroidism (≥60 ng/mL), anemia (Hb <11 g/dL), hyperphosphatemia (phosphate>1.38 mmol/L), acidosis (venous tCO_2_ <22 mmol/L), hyperkalemia (potassium >5 mmol/L).

Interestingly, at the first visit, improvers had already developed fewer metabolic complications than nonimprovers: 0.7±0.6 and 1.0±1.0, p = 0.02, respectively. They also developed fewer complications during follow-up: 0.5±0.6 and 1.2±0.8, p<0.0001. The prevalence of two or more metabolic complications at the first visit was 6.5% in improvers and 25.0% in nonimprovers (p = 0.0012); at the last visit, these percentages were 4.8% and 38.8% (p<0.001) respectively (Figure 2).

## Discussion

In this study, we demonstrate that renal function can improve sustainably over time in a subgroup of patients followed and treated for CKD. Given the reputedly relentless nature of CKD, the existence of our group of improvers may appear surprising, but the methods we used attest to its reality. First, we measured GFR in all patients, using ^51^Cr-EDTA renal clearance, a gold standard method to assess renal function [23]. Similar trends were obtained based on MDRD or CKD-EPI eGFR. Second, all subjects were outpatients, in stable condition, without any recent therapeutic modifications. Given the length of the follow-up, neither extracellular volume expansion nor hemodynamic changes can account for the repeated GFR improvement. The median of four annual visits in the two study groups rules out any transient renal improvement or iatrogenic bias. Third, and most importantly, we demonstrated that the observed increase in mGFR was associated with a decrease in the number of metabolic complications; such a decrease reflects a true improvement in mGFR.

The 15.3% prevalence of GFR improvement observed in this cohort is consistent with the few reports previously published. In the 2-year follow-up of the MDRD study, GFR remained stable in 19% of patients and improved in 11% [3]. In the AASK trial, however, over a longer period of 8.8 years and with Bayesian models, eGFR improved among only 3.3%, with a mean slope of +1.06 ml/min/1.73 m^2^ per year [19]. This study also emphasized that many patients with CKD have a nonlinear GFR trajectory or a prolonged period of nonprogression [24]. In a population with mild CKD receiving primary care through a large integrated health care system between 2004 and 2009, eGFR rose over time among 41.3% [25]. In a retrospective study of patients before nephrology referral, eGFR did not progress among 16% of those with stages 3–5 of CKD [26]. After referral, the eGFR decline slowed to less than 1 ml/min/1.73 m^2^/year in 55% of patients, including those with an improving slope. Others have emphasised the beneficial effect of nephrology referral and reported a positive slope for eGFR (more than +1 ml/min/1.73 m^2^/year) in 18% of patients in stages 2 and 3 of CKD and in 24% of those in stage 4 [26]. Most of those studies, however, used estimated GFR and did not describe in detail the features of the subgroup with this positive slope.

A second important finding is that this favourable disease course occurred in patients with various initial nephropathies. The notable exceptions were diabetic and polycystic kidney diseases, not seen in any improvers. Both those nephropathies are well known for their relatively poorer prognosis. Polycystic kidney disease has been reported to be resistant to ACE inhibitor treatment and to progress relentlessly even after inhibition of cyst development [27], [28]. Diabetic glomerulopathy is characterized by high urinary albumin excretion, a major negative prognostic factor, even though renal decline appears similar in diabetic and non-diabetic patients at comparable levels of albuminuria [29], [30]. Our data, like those of others, emphasize the importance of albuminuria as a prognostic factor of renal function. We should specify that the diagnosis of diabetic glomerulopathy in the NephroTest cohort was based on either renal biopsy or clinical data (diabetes, renal failure, high urinary albumin excretion, and/or other microvascular complications).

Surprisingly, GFR also improved in some patients with advanced CKD: 24.2% of the improvers had advanced CKD, in stage 4 or 5. Our results suggest that the ability to heal persists in some conditions in advanced CKD. This is consistent with previous evidence from the REIN study demonstrating that the tertile with the lowest GFR at inclusion had the most ESRD events prevented [31]. Mechanisms of this improvement in renal function remain unknown, but might involve renal tissue remodeling during follow-up, perhaps mediated by angiotensin II blockers. Certainly, more than 90% of our cohort takes ARBs or ACE (>90%), and they are known to induce regression of renal fibrosis in experimental models [32]–[34].

We assessed the number of achieved recommended treatment targets, based on the main established modifiable risk factors for CKD, including systolic and diastolic blood pressure, proteinuria or albuminuria, and use of angiotensin blockers. We observed a higher number of achieved targets in improvers than in nonimprovers at all visits, despite a lower need for antihypertensive and antiproteinuric treatments. The use of a higher number of treatments in nonimprovers may clearly reflect indication bias. However, it is important to note that the number of achieved targets was significantly associated with mGFR improvement independent of baseline or mean mGFR and of progression risk factors. Thus our results confirm the value of achieving recommended therapeutic targets to preserve renal function. Improvers also differed from nonimprovers in their CKD metabolic complications. This finding underlines the possible deleterious role of metabolic complications on GFR progression. We considered CKD metabolic complications to include hyperphosphatemia, metabolic acidosis, anemia, hyperkalemia, and elevated parathormone, as previously reported [21]. High serum phosphate has also been recognized as an independent risk factor for renal disease progression in several observational studies [35], [36]. In the REIN trial, patients with phosphate levels in the two highest quartiles progressed significantly faster to a composite endpoint of doubled serum creatinine or ESRD compared with patients whose phosphate levels were below the median [37]. Moreover, the renoprotective effect of ACE inhibitor decreased as serum phosphate increased. The authors hypothesized that fibroblast growth factor-23 (FGF-23) might activate the renin-angiotensin system (RAS).

Metabolic acidosis is another risk factor for renal disease progression, and its correction with sodium bicarbonate slows renal function decline in stage 2–5 CKD [38]. However, after adjusting for mGFR, we found no difference between groups for metabolic complications considered individually, with the notable exception of native vitamin D level We nonetheless cannot rule out the possibility that we lack the necessary statistical power.

We found that native vitamin D deficiency was less prevalent in improvers than nonimprovers. This result is important because vitamin D deficiency is very common in CKD patients, and its prevalence increases as GFR declines [39]. Although there is not yet any proof that an insufficient 25(OH)D level contributes to GFR impairment, a few studies have reported similar results. In a community-based cohort of ambulatory older adults, lower serum 25(OH)D concentrations were associated with faster eGFR loss, particularly when 25(OH)D was lower than approximately 30 ng/ml [40]. Moreover, lower levels of 25(OH)D were related to higher risks of ESRD and mortality in patients with stage 2–5 CKD [41]. In NHANES III, a 25(OH)D concentration <15 ng/ml was associated with an increased risk of incident ESRD in black subjects [42]. These findings suggest that effective treatment of CKD metabolic complications and 25(OH)D deficiency could limit GFR loss.

Major strengths of this study include the quality of the patient phenotype, including repeated GFR measures with a reference method, and the variety of nephropathy types. Its principal limitation is that data could be confounded by a regression to the mean (RTM) phenomenon. RTM is indeed a concern when the outcome is the evolution of a continuous variable over time. However, it is considerably reduced when the evolution is studied with several measurements, which we did by selecting patients with at least 3 measurements. Moreover, we performed several sensitivity analyses and found RTM was limited. In addition, our classification of patients was not based on mGFR slopes estimated from linear regression, but on the examination of mGFR trajectories by four independent nephrologists. While we cannot rule that this qualitative method may have slightly overestimated the number of improvers, it is outweighted by strong agreement between nephrologists in their evaluation. Another limitation is that this is an observational study, and observed associations cannot be presumed to be causal relations. Moreover, as with all studies of change in GFR, our study is subject to potential survival bias, which may result in overestimating the prevalence of mGFR improvement. Finally, despite careful data collection, we cannot exclude unrecognized therapeutic modifications for some patients that could be responsible for bias.

In conclusion, our data show that renal function can improve over time in a significant proportion of CKD patients, even at a severe stage. It is noteworthy that the observed GFR increase is associated with a decrease in the number of metabolic complications over time, thus demonstrating true renal function improvement. Achievement of the current targets of nephroprotection is essential for preserving renal function. Our results also suggest that treating metabolic complications and 25(OH)D deficiency more effectively could promote improvement in renal function. Prospective replication of these findings during intervention trials is now required.

## Supporting Information

File S1 **Combined file of supporting figures and tables.**
(DOC)
