# Supplementary material for: Renal Function Can Improve at Any Stage of Chronic Kidney Disease
Source: PLoS One. 2013 Dec 13;8(12):e81835. doi: 10.1371/journal.pone.0081835 (PMC3862566; doi:10.1371/journal.pone.0081835)
Supplement: File S1 — Combined file of supporting figures and tables. (DOC) [file pone.0081835.s001.doc]

**Legends**

**Figure 1S :** mGFR trajectories of patients classified as improvers and excluded for erratic curves. Trajectoires for improvers are divided depending of the number of GFR measurements

**Figure 2S:** Distribution of mGFR slopes for all patients, improvers and non-improvers

Figure 1S

**
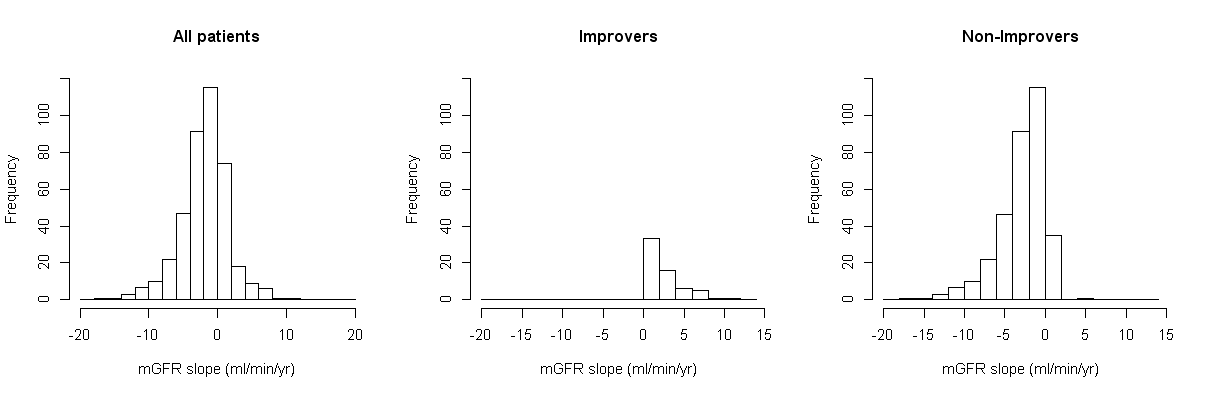
Figure 2S**

**Table 1S:** Evolution of antihypertensive treatments during follow-up

|  | **Improvers**  (N=59/62) | **Non improvers**  (N=332) | p-value |
| --- | --- | --- | --- |
| ACEi or ARB |  |  |  |
| *First visit* | 72.9(43/59) | 81.9(272/332) | 0.1 |
| *Last visit* | 81.4(48/59) | 92.2(306/332) | 0.009 |
| Loop diuretics |  |  |  |
| *First visit* | 35.6(21/59) | 39.5(131/332) | 0.6 |
| *Last visit* | 27.1(16/59) | 50.9(169/332) | 0.0007 |
| Number of different medications |  |  |  |
| *First visit* | 2.29 ± 1.22 | 2.51 ± 1.37 | 0.2 |
| *Last visit* | 2.42 ± 1.25 | 3.03 ± 1.41 | 0.002 |
| *Mean across visits* | 2.39 ± 1.13 | 2.82 ± 1.30 | 0.01 |
